# Structure-Activity–Dependent Regulation of Cell Communication by Perfluorinated Fatty Acids using *in Vivo* and *in Vitro* Model Systems

**DOI:** 10.1289/ehp.11728

**Published:** 2008-10-23

**Authors:** Brad L. Upham, Joon-Suk Park, Pavel Babica, Iva Sovadinova, Alisa M. Rummel, James E. Trosko, Akihiko Hirose, Ryuichi Hasegawa, Jun Kanno, Kimie Sai

**Affiliations:** 1 Department of Pediatrics and Human Development, National Food Safety and Toxicology Center, Michigan State University, East Lansing, Michigan, USA;; 2 Division of Risk Assessment and; 3 Division of Cellular and Molecular Toxicology, National Institute of Health Sciences, Tokyo, Japan

**Keywords:** extracellular receptor kinase, gap-junctional intercellular communication, mitogen-activated protein kinase, perfluorooctanoate, perfluoropentanoate, phosphatidylcholine-specific-phospholipase C, tumor promotion

## Abstract

**Background:**

Perfluoroalkanoates, [e.g., perfluorooctanoate (PFOA)], are known peroxisome proliferators that induce hepatomegaly and hepatocarcinogenesis in rodents, and are classic non-genotoxic carcinogens that inhibit *in vitro* gap-junctional intercellular communication (GJIC). This inhibition of GJIC is known to be a function of perfluorinated carbon lengths ranging from 7 to 10.

**Objectives:**

The aim of this study was to determine if the inhibition of GJIC by PFOA but not perfluoropentanoate (PFPeA) observed in F344 rat liver cells *in vitro* also occurs in F344 rats *in vivo* and to determine mechanisms of PFOA dysregulation of GJIC using *in vitro* assay systems.

**Methods:**

We used an incision load/dye transfer technique to assess GJIC in livers of rats exposed to PFOA and PFPeA. We used *in vitro* assays with inhibitors of cell signaling enzymes and antioxidants known to regulate GJIC to identify which enzymes regulated PFOA-induced inhibition of GJIC.

**Results:**

PFOA inhibited GJIC and induced hepatomegaly in rat livers, whereas PFPeA had no effect on either end point. Serum biochemistry of liver enzymes indicated no cytotoxic response to these compounds. *In vitro* analysis of mitogen-activated protein kinase (MAPK) indicated that PFOA, but not PFPeA, can activate the extracellular receptor kinase (ERK). Inhibition of GJIC, *in vitro*, by PFOA depended on the activation of both ERK and phosphatidylcholine-specific phospholipase C (PC-PLC) in the dysregulation of GJIC in an oxidative-dependent mechanism.

**Conclusions:**

The *in vitro* analysis of GJIC, an epigenetic marker of tumor promoters, can also predict the *in vivo* activity of PFOA, which dysregulated GJIC via ERK and PC-PLC.

Research on the environmental fate and toxicology of halogenated compounds has focused primarily on brominated and chlorinated organics, whereas fluorinated organics received less attention, partly because of the perception that these compounds, which are quite chemically inert, were also biologically inert (Key et al. 1997). However, perfluorinated fatty acids (PFFAs), such as perfluorooctanoate (PFOA) and perfluorooctane sulfonate (PFOS), are found in the environment and have been detected in the blood of animals throughout the world, including the seals of remote arctic regions, indicating widespread distribution (Kannan 2001; Tao 2006; Van de Vijver 2005). Significant levels of PFOA and PFOS have also been detected in the serum of humans, but there is evidence of a significant decline in body burdens of PFOS and PFOA over the last 5–10 years (Calafat et al. 2007). The values from the first National Health and Nutrition Examination Survey (NHANES) conducted from 1999 to 2000 reported geometric means of 30.4 μg PFOS/L and 5.4 μg PFOA/L, and the second NHANES conducted between 2003 and 2004 reported geometric means of 20.7 μg PFOS/L and 3.9 μg PFOA/L (Calafat et al. 2007). Contamination of the environment is not limited to PFOA and PFOS but also includes short-chain perfluorinated alkanoates, such as perfluorobutyrate, perfluoropentanoate (PFPeA), perfluorohexanoate, and perfluoroheptanoate (Skutlarek et al. 2006).

The acute toxicities of PFOA and PFOS in rodent systems are low (Hekster 2003; Kudo and Kawashima 2003). After the absorption of PFOA into the body, it is predominantly distributed in the liver and plasma and, to a lesser extent, the kidney and lungs (Kudo and Kawashima 2003). Thus, the chronic and short-term effects of PFOA in rats are found largely in the liver (Kennedy et al. 2004) and immune system (DeWitt et al. 2008). Peroxisome proliferation in rodent livers is one of the major responses to PFOA, along with subsequent interferences with normal metabolism of fatty acids and cholesterol, and the induction of hepatocellular hypertrophy (Kennedy et al. 2004). Peroxisome-proliferating chemicals are classic nongenotoxic tumor promoters in rodent liver tissue (Cattley et al. 1995), and like other peroxisome proliferators, PFOA has also been shown to strongly promote tumors in rodent livers (Abdellatif et al. 1991). However, peroxisome-proliferating compounds might not be strong tumor promoters in human livers because of species differences in the response to peroxisome proliferators *in vivo*, with rodents more responsive than primates (Klaunig et al. 2003).

Although the underlying mechanisms of tumor promotion might vary, such as the induction of peroxisome proliferation, tumorigenic cells have long been characterized as cells that lose their ability to regulate growth through contact inhibition (Borek and Sachs 1966) and lack the ability to terminally differentiate (Potter 1978), which implies a breakdown in one of the communicating mechanisms (Trosko and Upham 2005). Tumorigenic cells can be benign, leading to the compression of surrounding tissues, or have the potential to acquire genetic mutations that lead to a malignant state where the cancerous cells can invade surrounding tissues. Alteration of cell-to-cell communication via gap junctions has been implicated in the tumorigenic process and is supported by considerable evidence (Trosko and Ruch 2002).

Inhibition of gap-junctional intercellular communication (GJIC) appears to be a necessary, albeit insufficient, step of tumorigenesis and is therefore a common response of cells to tumor promoters, oncogenes, growth factors, and nongenotoxic carcinogens such as peroxisome proliferators (Trosko and Ruch 1998; Trosko and Upham 2005). Although GJIC is modulated by multiple signaling pathways, simple bioassays of intercellular communication can be used to assess dysregulation of gap junctions regardless of the upstream effectors. Thus, GJIC is an excellent biomarker first to assess the potential tumorigenicity of chemicals and then to use as a cell signaling end point to determine the early molecular events induced by these chemicals.

Cell proliferative diseases, such as cancer, not only require the release of a quiescent cell from growth suppression via down- regulation of GJIC and/or changes in extracellular components (i.e., integrins), but also need to activate mitogenic signaling pathways. The mitogen-activated protein kinase (MAPK) pathways are the major intracellular signaling mechanisms by which a cell activates, via phosphorylation, transcription factors involved in mitogenesis (Denhardt 1996). The extracellular receptor kinase (ERK) pathway has been extensively characterized, is the most understood of the MAPK pathways (Denhardt 1996), and is a key pathway of carcinogenesis (Roberts and Der 2007).

In the present study, we extended our *in vitro* studies with F344 rat liver epithelial cells, which determined that PFOA, but not PFPeA, inhibited GJIC (Upham et al. 1998), to an *in vivo* study using F344 rats exposed to PFOA, PFPeA, or phenobarbital (PB), a known tumor promoter, to determine GJIC in liver tissue. We also continued our *in vitro* studies of PFOA versus PFPeA in determining differential effects of these compounds on MAPK, specifically ERK, and further determined that the mechanism of PFOA-induced inhibition of GJIC depends on redox activity, ERK, and phosphatidylcholine-specific phospholipase C (PC-PLC).

## Materials and Methods

### Chemicals

**Figure 1:**
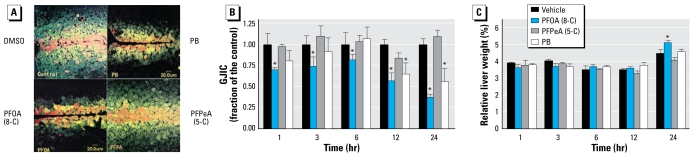
Analysis of *in vivo* effects of PFOA and PFPeA on GJIC in the liver tissue using IL/DT technique. Abbreviations: 5-C, five carbon; 8-C, eight carbon. (*A*) A fluorescent image of an IL/DT analysis of GJIC in the liver tissue of rats at 24 hr after a single i.p. administration of DMSO (vehicle), PB, PFOA, or PFPeA. Bar = 20 μm. (*B*) Mean + SD of the IL/DT data from rats treated with DMSO, PB, PFOA, or PFPeA for the acute exposure group. (*C*) Mean + SD relative liver weight from rats treated with DMSO, PB, PFOA, and PFPeA for the acute exposure group. *p < 0.05 compared with vehicle, determined by one-way ANOVA for each time group followed by Dunnett’s post hoc test.

**Figure 2:**
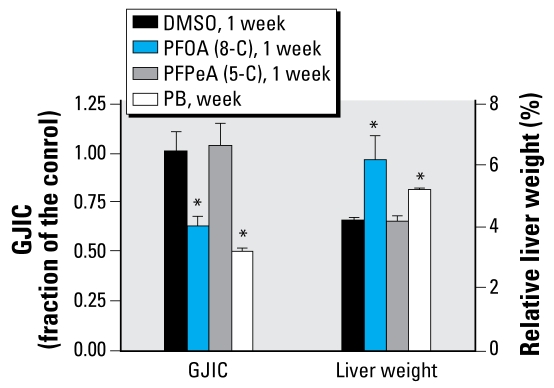
The long-term effects (1 week) of PB, PFOA, and PFPeA on GJIC and RLW (mean + SD). Abbreviations: 5-C, five carbon; 8-C, eight carbon. A one-way ANOVA was done for the GJIC data and a Kruskal-Wallis one-way ANOVA was done for the RLW data because these data failed the normality test. *p **<** 0.05 compared with vehicle (DMSO); significant effects determined by ANOVA or Kruskal-Wallis ANOVA for each group was followed with a Dunnett’s post hoc test at *p* < 0.05.

**Figure 3:**
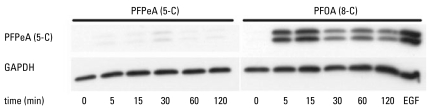
Activation of ERK-MAPK by PFOA, but not by PFPeA, in F344 WB rat liver epithelial cells determined by Western blots: Top panel probed with a phosphorylated ERK specific antibody and the bottom panel probed with a GAPDH specific antibody. The concentrations of PFPeA and PFOA were 100 μM. The concentration and time of incubation for epidermal growth factor (EGF) was 20.0 ng/mL and 15 min.

**Figure 4:**
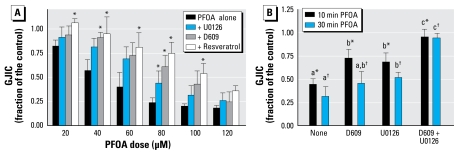
(*A*) Prevention of PFOA-induced inhibition of GJIC by inhibitors of MEK and PC-PLC and resveratrol at various doses of PFOA (mean + SD). The concentrations and times of preincubation of U0126, D609, and resveratrol were 20 μM/30 min, 50 *μM/20 min, and 100 μM/15 min, respectively. A one-way ANOVA was done for each dose group. *Significant at *p* < 0.05 using the Dunnett’s post hoc test that compared each inhibitor treatment with that of PFOA alone. (*B*) The interactive effect of MEK and PC-PLC inhibitors on reversing PFOA-induced inhibition of GJIC at 10 and 30 min (mean + SD). The concentrations and times of preincubation of U0126 and D609 were 20 μM/30 min and 50 μM/20 min, respectively. A one-way ANOVA indicated significance at *p* < 0.05 for each time group. The Tukey pairwise-comparison post hoc test was used to determine statistical differences, as indicated by different letters, between the inhibitor treatments for each time group. The lettered asterisks represent the 10-min group and lettered daggers represent the 30-min group.

**Figure 5:**
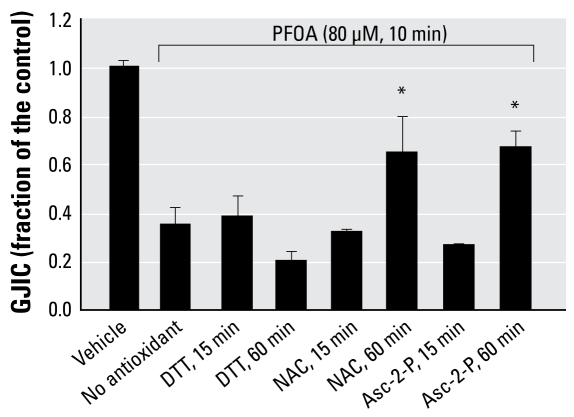
Prevention of PFOA-induced inhibition of GJIC by various antioxidants (mean + SD). The concentrations of PFOA, DTT, Nac, and Asc-2-P were 80 μM, 10 mM, 100 μM, and 100 μM, respectively. *p < 0.05 by ANOVA and Dunnett’s post hoc test comparing each antioxidant treatment with that of PFOA alone (no antioxidant).

**Figure 6:**
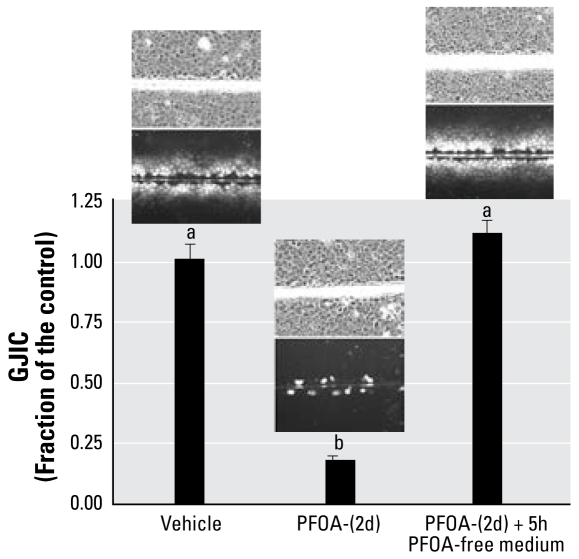
The effects of an extended incubation of cells with PFOA (80 μM, 2 days) and transfer of cells to PFOA-free medium (5 hr) on cell morphology and GJIC (mean + SD). Each phase-contrast and fluorescent photomicrograph represents one of the three replicates of each treatment group (magnification, 200×). Different letters indicate significance at *p* < 0.05 using ANOVA and Tukey post hoc test with a pairwise comparison.

We purchased PFOA (purity > 90%) and PFPeA (purity = 97%), for the data presented in Figures 1–3 and 4A, from Fluka Chemie AG (Buchs, Switzerland), and because of unavailability from Fluka, we purchased PFOA for the data presented in Figures 4B, 5, and 6 from Aldrich Chemical Company Inc. (Milwaukee, WI, USA), with a purity of 96%. The purity values were obtained from the commercial sources. The ratios of linear versus branched isomers in our samples were undetermined. The stock solutions were prepared by dissolving the powder in the solvent: acetonitrile for the *in vitro* assays and dimethyl sulfoxide (DMSO) for the *in vivo* studies; we also used these solvents as the vehicle controls. We purchased Lucifer yellow (LY) from Molecular Probes (Eugene, OR, USA); sodium dodecyl sulfate, Tween 20, Tris, glycine, acrylamide, tetramethylethylenediamine (TEMED) and DC protein kit from Bio-Rad Laboratories (Hercules, CA, USA); DMSO, rhodamine-dextran (RhD; molecular weight, 10,000 Da), dithiothreitol (DTT), *N*-acetylcysteine (Nac), l-ascorbate-2-phosphate (Asc-2-P) sesquimagnesium salt hydrate, and PB from Sigma-Aldrich Chemical Company (St. Louis, MO, USA); D609 and U0126, from Tocris Bioscience (Ellisville, MO, USA); resveratrol from CTMedChem (Bronx, NY, USA); acetonitrile, from EM Science (Gibstown, NJ, USA); polyclonal antibodies directed to phospho-ERK, from New England Biolabs (Ipswich, MA, USA); and mouse polyclonal antibody directed to glyceraldehyde 3-phosphate dehydrogenase (GAPDH), from Chemicon (Temecula, CA, USA).

### *In vivo* study

#### Animal treatment

The protocol for this study was approved by the Animal Care and Utilization Committee of the National Institutes of Health Sciences of Japan to assure that the rats were treated humanely and with regard for alleviation of suffering. Male Fischer-344 (F344) rats, 5 weeks old, were purchased from Charles River Japan (Kanagawa, Japan) and housed in plastic cages (five rats/cage). Male F-344 rats were chosen to match the *in vitro* studies that used liver epithelial cells isolated from male F-344 rats. The rats were kept under conditions of controlled temperature (23 ± 2°C), humidity (55 ± 5%), and lighting (12/12-hr dark/light cycle) and given CRF-12 basal diet (Oriental Yeast Co., Tokyo, Japan) and tap water *ad libitum*.

We used the rats in the experiments after 1 week of acclimation. Eighty rats were divided into four groups and twenty rats per group were treated with a single intraperitoneal (i.p.) administration of 100 mg/kg PFOA, 100 mg/kg PFPeA, 100 mg/kg PB, or only vehicle (DMSO). Four rats per group were killed under anesthesia at 1, 3, 6, 12, and 24 hr after administration. Another 16 rats were divided into four groups and four rats of each group were given powder diet containing PFOA, PFPeA, PB, or basal powder diet only (control), and then killed after 1 week. The diets were prepared by blending each chemical into the basal powder diet at final concentrations of 0.02% for PFOA and PFPeA and 0.05% for PB. We determined the weight of the rats at the beginning and end of the experiment, and the food consumption on days 3 and 7 of the experiment. Based on the average weight of the rats and the average food consumed per day, the estimated daily doses of chemical exposures for PFOA, PFPeA, and PB were 37.9, 32.3, and 93.3 mg/day/kg, respectively.

Diethyl ether was used to euthanize the rats. Before sacrifice, blood was collected from the orbital venous plexus under anesthesia with diethyl ether and prepared for measuring serum aspartate aminotransferase (sAST), serum alanine aminotransferase (sALT), and serum alkaline phosphatase (sALP). Determination of sAST, sALT, and sALP was carried out with a Hitachi automatic Analyzer 7150 (Hitachi, Ltd., Tokyo, Japan) using commercially available GOP, GPT and ALP diagnostic reagents (Wako Pure Chemical Industries, Ltd., Tokyo, Japan). After opening the abdominal cavity, we excised the liver and immediately used one part of the liver for the incision loading/dye transfer (IL/DT). Our preliminary study confirmed that the anesthetic and the vehicle, DMSO, under our experimental conditions did not affect *in vivo* GJIC.

#### Bioassay of GJIC (IL/DT)

We assayed *ex vivo* GJIC in the liver by the IL/DT method described previously (Sai et al. 2000). A part of the left lobe of the liver was put on a plastic plate covered with wet gauze. A mixture of fluorescent dyes containing 0.5 mg/mL LY and 0.5 mg/mL RhD in phosphate-buffered saline (PBS) was dropped on the tissue’s surface. Three to four incisions were made on the surface of each specimen with a sharp blade. Excess amount of dye mixture was additionally put into the incisions and kept there for 3 min at room temperature. After incubation, the tissue was washed with PBS three times and fixed in 10% phosphate-buffered formalin overnight. Slices were washed with water and processed for embedding in paraffin. Five μm sections for GJIC analysis were prepared by cutting the paraffin block perpendicular to the incision line. Areas stained with LY alone or with RhD were detected by the emission of fluorescence using a confocal microscope (Fluoview, Olympus, Tokyo, Japan). We counted the number of cells stained with LY alone and normalized this number by dividing by the incision length. At least three incision sites per specimen were randomly chosen for the analysis, and the mean value was used as data from one animal. The values were expressed as a fraction of the control.

### *In vitro* study

#### Cell culture

We obtained the WB-F344 rat liver epithelial cell line from J.W. Grisham and M.S. Tsao of the University of North Carolina at Chapel Hill, Chapel Hill, NC, USA (Tsao et al. 1984). Cells were cultured in D-medium (formula 78–5470EF, Gibco Laboratories, Grand Island, NY, USA), supplemented with 5% fetal bovine serum (Gibco Laboratories), and incubated at 37°C in a humidified atmosphere containing 5% CO_2_ and 95% air. The cells were grown in 35-mm tissue culture plates (Corning Inc., Corning, NY, USA) and the culture medium was changed every other day. Bioassays were conducted with confluent cultures that were obtained after 2–3 days of growth.

These WB cells are diploid and nontumorigenic (Tsao et al. 1984) and have been extensively characterized for GJIC in the absence and presence of well-known tumor promoters, growth factors, tumor suppressor genes, and oncogenes (Trosko and Ruch 1998). Intrahepatic transplantation of WB cells, which are liver bipolar stem cells, into adult syngenic F344 rats results in the morphologic differentiation of these cells into hepatocytes and incorporation into hepatic plates (Coleman et al. 1993).

#### Bioassay of GJIC (scrape load/dye transfer)

The scrape loading/dye transfer (SL/DT) technique was adapted after the method of Upham et al. (1998). The test chemicals were added directly to the cell culture medium from concentrated stock solutions. The migration of the dye through gap junctions was visualized with a Nikon Eclipse TE3000 phase contrast/fluorescent microscope and the images were digitally captured with Nikon EZ Cool Snap charge-coupled device camera (Nikon Inc., Nikon, Japan). GJIC was assessed by comparing the distance the dye traveled in the chemically treated cells with the distance the dye traveled in the vehicle controls, which was measured using the Gel-Expert imaging software (Nucleotech, San Mateo, CA, USA). We report GJIC as a fraction of the control. Based on previous results (Upham et al. 1996, 1998), 1-methylanthracene as well as PFOA were used as positive controls of inhibition of GJIC, whereas acetonitrile at vehicle concentrations was used as a negative control. The vehicles used for the *in vitro* assays, acetonitrile and PBS, had no effect on GJIC. We performed all experiments at least in triplicate and report the results as means ± SD at the 95% confidence interval.

### Western blot analysis

Cells were grown in 35-mm-diameter Corning tissue culture plates to the same confluency as the SL/DT assay. The cells were depleted of serum 5 hr before addition of PFFAs to synchronize the cells into G_0_ to minimize background ERK levels. This does not alter the effect on GJIC in the F344 WB cells, as previously determined (Rummel et al. 1999). The proteins were extracted with 20% sodium dodecyl sulfate (SDS) solution containing 1 mM phenylmethylsulfonyl fluoride, 100 μM Na_3_VO_4_, 100 nM aprotinin, 1.0 μM leupeptin, 1.0 μM antipain, and 5.0 mM NaF. The protein content was determined with the Bio-Rad DC assay kit. The proteins were separated on 12.5% SDS–polyacrylamide gel electrophoresis according to the method of Laemmli (1970). Fifteen micrograms protein was loaded onto the gels and electrophoretically transferred from the gel to polyvinyl difluoride membranes (Millipore Corp., Bedford, MA, USA). Phosphorylated ERK 1 and ERK 2 were detected with a 1:2,000 dilution of anti-phospho-ERK polyclonal antibodies, and GAPDH was detected with a 1:10,000 dilution of anti-GAPDH polyclonal antibodies, that were incubated sequentially with the membranes, each for 2 hr. The protein–primary antibody complex was probed with a 1:1,000 dilution of horseradish peroxidase–conjugated anti-rabbit or anti-mouse antibodies (Amersham Life Science Products, Arlington Heights, IL, USA) for 1 hr. The ERK and GAPDH protein bands were detected using the Super Signal chemiluminescence detection kit (Pierce Corp., Arlington Heights, IL, USA), enhanced chemiluminescence (ECL) detection kit, and ECL Hyperfilm–MP (Amersham Life Science Products, Denver, CO, USA).

### Statistics

For the *in vivo* studies, the value of each group was expressed as the mean ± SD of data derived from four rats. The *in vitro* assays were done in at least triplicate and expressed as a fraction of the control. The significance of differences in all results was evaluated with either a one-way analysis of variance (ANOVA) or, if the data set failed the normality test, a Kruskal-Wallis one-way ANOVA on ranked means. Normality assumption testing was done with the Kolmogorov-Smirnov test and equal variance assumption testing with the Levene median test. If ANOVA or Kruskal-Wallis ANOVA rejected the null hypothesis, then the results that were compared with a designated control used Dunnett’s multiple-comparison post hoc tests or Tukey’s post hoc test for pairwise multiple comparisons.

## Results

### *In vivo* results

The *in vivo* results of PFOA and PFPeA were compared with PB, a known liver tumor promoter. We used two different dosing schemes: an acute 24-hr exposure via i.p. administration and a longer-term (1 week) dietary exposure. An ANOVA indicated that PFOA, PFPeA, and PB had no statistically significant effect on body weights of the rats (data not shown). Liver injury was assessed using the biomarkers sALT, sAST, sALP, and the results for both dosing schemes are presented in Table 1. At day 7, there were no significant differences between the rats treated with PFOA, PFPeA, and PB for all three of the selected liver enzymes, indicating no long-term liver injury. After 1 day, we found a small, biologically insignificant, but statistically significant increase in sAST, with the data exhibiting high variability.

To assess the *in vivo* effects of these compounds on GJIC in the liver tissue, we used an IL/DT technique. Figure 1A shows the incorporation of the fluorescent dye into the liver cells and subsequent distribution of the fluorescent dye through the gap junctions of the tissue. RhD, which is a large-molecular-weight dye that does not traverse gap junctions, is color-coded red. LY, which does travel through gap junction channels, is color-coded from yellow for high intensity to green for lower intensity. We measured and averaged the distances traveled by the gap-junction–permeable dye and show them in Figure 1B (acute exposure) and Figure 2 (long-term exposure). PFOA and PB but not PFPeA inhibited *in vivo* GJIC in the liver tissues of rats treated either acutely or chronically. Significant inhibition of GJIC by PFOA was observed after 1 hr, and continued to inhibit GJIC until 24 hr in the acutely treated rats. Significant inhibition of GJIC did not begin until after 12 hr of treatment with PB in this group of rats.

In the acute dose regimen (Figure 1C), a significant increase in the relative weight of livers from rats treated with PFOA was observed at 24 hr. Similarly, rats chronically exposed to PFOA and PB for 1 week had significant increases in relative liver weight (RLW; Figure 2). The livers of animals treated either acutely or chronically with PFPeA did not significantly increase in relative weights compared with rats fed the vehicle (Figures 1C, 2).

### *In vitro* results

Considering that the *in vitro* results of PFOA and PFPeA effects on gap junctions correlated with their effects on gap junctions *in vivo*, we did further *in vitro* analyses of PFOA to determine underlying mechanisms involved in the dysregulation of GJIC. PFOA, which inhibits GJIC, also activated ERK as determined by Western blot analysis of the phosphorylated, activated form of ERK (Figure 3). In contrast, the non-GJIC inhibitory PFPeA did not activate ERK (Figure 3). Activation of ERK was within 5 min in cells treated with PFOA, which correlates with the time of inhibition of GJIC, indicating a potential link. Preincubation of the cells with an MEK inhibitor, U0126, partially but significantly prevented the inhibition of GJIC by PFOA (Figure 4A). Preincubation of the cells with the PC-PLC inhibitor D609 also partially but significantly prevented the inhibition of GJIC by PFOA (Figure 4A). The significant contribution of PC-PLC and MEK in PFOA-induced inhibition of GJIC diminished after the maximum inhibitory dose of 80 μM to a nonsignificant involvement at the higher dose of 120 μM (Figure 4A), indicating further that mechanisms other than MEK and PC-PLC are also involved.

Gap junctions are known to be redox sensitive, so we conducted several experiments with various antioxidants. Resveratrol significantly reversed the inhibitory effect on GJIC and was possibly inhibiting both MEK and PC-PLC (Figure 4A). Additional experiments were performed to look at the combinatorial effect of pretreating cells with both D609 and U0126. The combination of both of these inhibitors of signal transduction enzymes resulted in the prevention of GJIC inhibition by PFOA, and the combinatorial effect was significantly greater than cells treated with either inhibitor alone as determined by a Tukey post hoc multiple-comparison test (Figure 4B). These results collectively indicate that PFOA-induced regulation of GJIC is a function of both of these signaling enzymes.

Further experiments were performed with DTT, Nac, and Asc-2-P (Figure 5). DTT and Nac in the absence of PFOA had no statistically (ANOVA) significant effect on GJIC at both 15 and 60 min (data not shown). Asc-2-P had a small, < 10% effect (ANOVA, Tukey) on GJIC in the absence of PFOA at 15 min but not 60 min (data not shown). Asc-2-P and Nac both prevented the inhibition of GJIC by PFOA within a 60-min pre-incubation time, but not DTT, implicating redox-sensitive proteins that probably do not involve thiol oxidations. Preincubation of Asc-2-P and Nac for 15 min did not reverse the effect of PFOA on GJIC. The oxidative nature of PFOA was not cytotoxic, as indicated after 2 days of growing cells after the log-phase of growth with 80 μM PFOA, resulting in no visual abnormalities in the morphology of the cells and complete restoration of GJIC after the cells were transferred to fresh medium for 5 hr containing no PFOA (Figure 6).

## Discussion

Understanding the biological effects of the environmentally prevalent PFFAs on cell signaling pathways relevant to the epigenetic, nongenotoxic phase of cancer is important. In particular, GJIC offers a very central signaling system to assess risk (Trosko and Upham 2005). Although the transient closure of gap junction channels during proliferation is a normal response to mitogens, the chronic inhibition of GJIC by toxicants and toxins or by cytokines released during compensatory hyperplasia could lead to pathologic states (Trosko and Upham 2005; Upham and Trosko 2006). Thus, we conducted two dosing schemes, one a short term of 24 hr following an i.p. injection of PFOA, PFPeA, or PB, and another a longer-term study where the rats were dosed with these compounds through their daily feedings for 1 week. We previously demonstrated that inhibition of GJIC using *in vitro* model systems by perfluoroalkyl carboxylates and sulfonates depended on the chain length, where PFFAs with 7–10 carbons inhibited GJIC, and PFFAs with 2–6 carbons did not (Hu et al. 2002; Upham et al. 1998). To determine if chain length of PFFAs would exhibit similar effects on GJIC in a living organism, we treated F344 rats with PFOA, an eight-carbon PFFA, and PFPeA, a five-carbon PFFA, and determined GJIC in the liver tissue using an *ex vivo* IL/DT assay.

The liver is the primary target of PFOA (Kudo and Kawashima 2003), which is known to induce hepatocellular tumors in rodent model systems (Abdellatif et al. 1991; Kennedy et al. 2004). Similar to our *in vitro* results (Hu et al. 2002; Upham et al. 1998), PFOA decreased GJIC activity in the liver compared with the rats treated with the vehicle (control) for both the acute and long-term dosing schemes. In contrast, PFPeA-treated rats did not have altered GJIC in the livers compared with the control rats for both dosing schemes, which is also consistent with our *in vitro* observations. Another possible reason for the lack of an *in vivo* response by PFPeA could be a consequence of a greater elimination rate that is typical of PFFAs with shorter chain lengths (Chang et al. 2008; Ohmori et al. 2003). Although we did not measure the elimination rates of PFPeA in our experiments, the half-life of perfluorobutyrate is 9.2 hr (oral) and 6.4 hr (intravenous) in Sprague-Dawley rats (Chang et al. 2008). These half-lives are similar to that of PB in Sprague-Dawley rats, which is 8–9 hr. Considering that PB inhibited GJIC and induced hepatomegaly in the livers of the rats used in our experiments, and PFPeA did not inhibit GJIC using an *in vitro* assay system, we would expect that the noninhibitory effects of PFPeA on GJIC *in vivo* would not result from its increased rate of elimination. Further experiments are needed to confirm such a conclusion.

We previously published data that indicated the treatment of Sprague-Dawley rats with PFOS resulted in a decrease in GJIC activity in the liver tissue; thus, PFOA and PFOS have similar activities (Hu et al. 2002). The following are additional reports demonstrating that tumor promoters, known to inhibit GJIC *in vitro*, also inhibited GJIC *in vivo*: pentachlorophenol (Sai et al. 2000), 2-acetylaminofluorene (Krutovskikh et al. 1991), PB (Kolaja et al. 2000; Krutovskikh 1995), polychlorinated biphenyls (Kolaja et al. 2000; Krutovskikh 1995), pregnenolone-16α-carbonitrile (Kolaja et al. 2000), cadmium (Jeong et al. 2000), clofibrate, and DDT (Krutovskikh 1995). Another interesting report on the *in vivo* effects of chemicals on GJIC is the treatment of rats with the antioxidants lycopene and alpha and beta carotene. High doses of these antioxidants resulted in a decrease in GJIC activity, whereas rats exposed to low doses exhibited an increase in GJIC (Krutovskikh et al. 1997). Although *in vivo* assessment of intercellular communication has been limited in both the number of studies and choice of organ, namely, the liver, these results, including those presented in this report, nevertheless suggest that the *in vitro* rat liver epithelial cell assay system is a good predictor of the *in vivo* effects of chemicals on gap junctions in the liver tissues of rodents.

PFOA and PB induced hepatomegaly, whereas PFPeA had no effect. These results are similar to those previously published indicating that PFOA, but not perfluorobutyrate, affected RLWs in F344 rats (Takagi et al. 1991). Although not causally linked, hepatomegaly has been correlated with the promotion of liver tumors by many peroxisome proliferator-activated receptor α agonists, including PFOA (Takagi et al. 1992). The null effect of PFPeA on GJIC and hepatomegaly suggest that PFPeA would not be a tumor promoter; however, two-stage (initiation and promotion) carcinogenesis studies would be needed to confirm this conclusion. Tissue necrosis is known to induce compensatory hyperplasia that leads to increased liver weights, but this is unlikely the cause of hepatomegaly in the PFOA- and PB-treated rats, considering that no visual damage of the liver was seen in the histologic sections (data not shown) and there was no increase in serum enzymes.

Tissue homeostasis in multicellular organisms depends on functional GJIC, and the disruption of intercellular communication has been linked to many diseases (Trosko and Upham 2005). PFOA clearly interrupted GJIC in the liver tissues of rats, but further experiments would need to be done in other species. PFOS also inhibited GJIC in rat liver tissue as well as *in vitro* systems that included dolphin kidney cells (Hu et al. 2002). Thus, the potential for cross-species effects of PFOA on GJIC implicates a health risk to multicellular organisms. Future experiments, particularly with human cell lines, will aid in determining differences in the sensitivity of various organisms to the effects of PFOS and PFOA on GJIC and allow for more accurate assessment of risks these compounds pose to humans and wildlife.

Considering that *in vitro* analyses of PFFA, using rat liver epithelial cells, accurately predicted the *in vivo* effects on GJIC for various PFFAs, we did further *in vitro* analyses of PFPeA- and PFOA-treated rat liver epithelial cells to determine potential signaling mechanisms involved in PFOA-induced regulation of GJIC. Connexin 43 (Cx43) is a phosphoprotein, and the phosphorylation of the carboxy terminus by protein kinases, such as protein kinase C (PKC), Src, and MAPKs, in the regulation of GJIC has been well documented (Solan and Lampe 2005). Although phosphorylation of gap junctions is known to regulate the function, assembly, internalization, and degradation of this protein complex, the alteration of connexin phosphorylation by protein kinases, such as MAPKs, does not necessarily dysregulate gap junction function (Hossain et al. 1999), nor does the activation of protein kinases (i.e., MAPK) alter the phosphorylation status of connexins (Upham et al. 2008).

This was also true for PFOA, which clearly activated ERK-MAPK (Figure 3) but did not induce a change in the phosphorylation pattern of Cx43 as previously determined by Western blot analysis (Upham et al. 1998). Whether or not gap junctions are phosphorylated, several compounds (i.e., growth factors, lindane, lysophosphatidic acid, 12-*O*-tetra-decanoylphorbol-13-acetate, and cannabinoids) are known to inhibit GJIC through a MEK-dependent pathway (Komatsu et al. 2006; Mograbi et al. 2003; Rivedal and Opsahl 2001; Upham et al. 2003). Although many compounds activate MAPKs, such as p38 and ERK, the mechanism of inhibiting GJIC by many of these compounds is independent from these MAPKs (Machala et al. 2003; Upham et al. 2008).

Our results indicated that PFOA activated ERK in F344 WB rat liver epithelial cells within 5 min, and this time period is within the interval required for the inhibition of GJIC by PFOA in this cell line. PFPeA, which does not inhibit GJIC in this cell line (Upham et al. 1998), also did not activate ERK. Preincubation of these cells with an MEK inhibitor, U0126, partially prevented PFOA from inhibiting GJIC, indicating that PFOA-induced modulation of GJIC was not solely dependent on the ERK pathway.

Recently, PC-PLC has been implicated in the dysregulation of GJIC in response to toxicants that regulate GJIC through an MEK-independent mechanism (Machala et al. 2003; Upham et al. 2008). Preincubation of F344 WB cells with the PC-PLC inhibitor D609 also partially prevented PFOA from inhibiting GJIC. These results suggest that PFOA is regulating GJIC through multiple cellular mechanisms. This becomes more apparent as the dose of PFOA is increased resulting in the inhibition of GJIC at a high dose of 120 μM that depended on neither PC-PLC nor MEK. However, maximum inhibition of GJIC by PFOA, which was around 80 μM, was very dependent on the activity of both MEK and PC-PLC. This was further apparent from the experiment where cells were pre-treated with a combination of both D609 and U0126, resulting in almost complete recovery of GJIC. The activation of ERK and PC-PLC will not only control gap junction function but is known to alter gene expression, leading to various pathologies, including cancer. The function of PC-PLC in tumorigenesis has not been extensively studied, yet there are significant reports indicating that PC-PLC does play a very significant role in cancer (Cheng et al. 1997). The ERK pathway has been extensively characterized and is the most understood of the MAPK pathways (Denhardt 1996) and is a key pathway of carcinogenesis (Roberts and Der 2007).

PFOA, but not perfluorobutyrate, is known to induce oxidative stress in the livers of rats, as indicated by 8-hydroxydeoxy-guanosine formation (Takagi et al. 1991), and redox mechanisms are known to commonly play a role in gap junction function (Upham and Trosko 2009). These oxidative signaling effects could be site-directed redox regulations of specific regulatory proteins or from general oxidative effects (Upham and Trosko 2008). Recently, we reported that the antioxidant resveratrol prevented inhibition of GJIC by dicumylperoxide but not by benzoylperoxide (Upham et al. 2007). Dicumylperoxide, but not benzoylperoxide, inhibits GJIC through a PC-PLC–dependent mechanism (Upham et al. 2007). Similar to dicumylperoxide, we showed that resveratrol prevented inhibition of GJIC by PFOA to a greater level than either D609 or U0126 alone, but similar to the level of GJIC recovery seen when cells were pretreated with both D609 and U0126. These results indicate the possibility that PFOA dysregulates GJIC through both MEK and PC-PLC and that protection of GJIC by resveratrol is potentially through oxidative signaling events controlling both MEK and PC-PLC. Beyond the implication of redox mechanisms of the resveratrol experiment, this antioxidant is regularly consumed by humans and is found in high concentrations in red wine and peanut products (Sobolev and Cole 1999; Wang et al. 2002), and thus may have some relevance to the health of humans that may be exposed to environmental toxicants, such as PFOA. Chemopreventive effects of resveratrol are known to inhibit initiation, promotion, and progression of tumors (Signorelli and Ghidoni 2005). Thus, resveratrol could potentially contribute to a protective effect in humans exposed to PFOA by significantly blocking PFOA from inhibiting GJIC.

The addition of Asc-2-P or Nac partially reversed the inhibitory effects of PFOA on GJIC, similar to that of resveratrol. In contrast, DTT did not prevent PFOA from inhibiting GJIC, indicating that the oxidative events controlling PC-PLC and Mek are not thiol based. The exposure of F344 WB cells to PFOA for 2 days showed no adverse effects on cell morphology, and they communicated normally after PFOA was removed from the medium (Figure 6), which implicates that the PFOA-induced oxidative events are not killing the cells. These results suggest that general oxidative processes are involved in PFOA-induced inhibition of GJIC and that health benefits could potentially be attained by the consumption of many antioxidant rich foods, particularly in individuals deficient in antioxidants. Moreover, the reversible properties of PFOA-induced inhibition of GJIC are consistent with the known reversible nature of tumor promoters in two-stage carcinogenesis model systems (Trosko and Upham 2005). These results also indicate that reversing the effect of PFOA on GJIC after a simple washing of the treated cells with PBS demonstrates that PFOA is not covalently or tightly bound to the cell. The effect of PFOA on GJIC was probably not a consequence of directly interacting with the gap junction proteins because the inhibition of MAPK and PC-PLC both prevented the GJIC effect. Possibly PFOA interacted with these two proteins or interacted with a signaling protein or receptor even further upstream.

In conclusion, the *in vitro* assay system used to assess the effects of PFOA and PFPeA on GJIC predicted the *in vivo* results of GJIC from rats treated with these compounds. GJIC plays a vital role in maintaining tissue homeostasis, and disruption of gap junction function can lead to diseased states such as tumorigenesis. These results are similar to other tumor-promoting compounds tested in both an *in vitro* and *in vivo* assay system. Although there are several mechanisms by which environmental compounds might promote an initiated cell, such as through peroxisome proliferator activated receptors or protein kinase C, the disruption of normal intercellular communication is an essential event of multiple tumorigenic mechanisms (Trosko and Upham 2005) and serves as a central biomarker to assess the epigenetic toxicity of contaminants (Rosenkranz et al. 1997; Trosko and Upham 2005), as well as to assess the potential anti-tumorigenic health benefits of nutrition based food products (Trosko and Upham 2005).

## Figures and Tables

**Table 1:** The effect of PFOA, PFPeA, and PB on the levels of various biomarkers of liver injury in F344 rats.

	Enzyme activity (mU/mL)			
Exposure, time, enzyme	DMSO (vehicle)	PFOA	PFPeA	PB
Acute (24 hr)				
sALT	51.5 ± 3.2	138.6 ± 126.4	56.3 ± 13.2	54.5 ± 4.1
sAST	98.8 ± 8.8	232 ± 169.8*	113.0 ± 17.6	100.6 ± 15.1
sALP	1672.8 ± 90.0	1521.8 ± 220.2	1495.0 ± 233.8	1561.0 ± 115.2
Longer-term (1 week)				
sALT	39.3 ± 2.0	41.2 ± 1.9	39.8 ± 3.0	39.7 ± 2.6
sAST	71.2 ± 10.0	70.4 ± 4.3	73.9 ± 10.7	76.1 ± 9.4
sALP	1488.8 ± 62.9	1394.5 ± 59.4	1449.5 ± 36.6	1349.3 ± 53.0

To determine significant effects, we performed a one-way ANOVA for sALP (1 day), sALP (1 week), and sALT (1 week) and a Kruskal-Wallis one-way ANOVA on ranks for sAST (1 day), sAST (1 week), and sALT (1 day). Any significant effects determined by ANOVA were followed by a Dunnett’s post hoc test, with DMSO designated as the control.

* *p* < 0.05.
